# Characteristics of mental health literacy measurement in youth: a scoping review of school-based surveys

**DOI:** 10.1007/s44192-024-00079-0

**Published:** 2024-07-18

**Authors:** Emma C. Coughlan, Lindsay K. Heyland, Ashton Sheaves, Madeline Parlee, Cassidy Wiley, Damian Page, Taylor G. Hill

**Affiliations:** 1 Department of Psychology and Neuroscience, Dalhousie University, 1355 Oxford St, Halifax, NS B3H 4J1 Canada; 2 Department of Psychology, Mount Saint Vincent University, Halifax, Canada; 3 Department of School and Clinical Child Psychology, Ontario Institute for Studies in Education at University of Toronto, Toronto, Canada; 4 University of Dundee, Dundee, UK

**Keywords:** Mental health literacy, Youth, School mental health services, Scoping review, Survey measurement

## Abstract

Mental health literacy (MHL) was introduced 25 years ago as knowledge and beliefs about mental disorders which aid in their recognition, management, or prevention. This scoping review mapped the peer-reviewed literature to assess characteristics of secondary school-based surveys in school-attending youth and explore components of school-based programs for fostering MHL in this population. The search was performed following the method for scoping reviews by the Joanna Briggs Institute (JBI). Searches were conducted in four scientific databases with no time limit, although all sources had to be written in English. Primary studies (*N* = 44) provided insight into MHL surveys and programs for school-attending youth across 6 continents. Studies reported that most youth experience moderate or low MHL prior to program participation. School-based MHL programs are relatively unified in their definition and measures of MHL, using closed-ended scales, vignettes, or a combination of the two to measure youth MHL. However, before developing additional interventions, steps should be taken to address areas of weakness in current programming, such as the lack of a standardized tool for assessing MHL levels. Future research could assess the feasibility of developing and implementing a standard measurement protocol, with educator perspectives on integrating MHL efforts into the classroom. Identifying the base levels of MHL amongst school-attending youth promotes the development of targeted programs and reviewing the alignment with program components would allow researchers to build on what works, alter what does not, and come away with new ways to approach these complex challenges, ultimately advancing knowledge of MHL and improving levels of MHL.

## Introduction

The concept of mental health literacy (MHL), originally defined by Jorm and colleagues [43] as “knowledge and beliefs about mental disorders which aid their recognition, management, or prevention” (p. 182), was developed to consider people’s level of knowledge of mental health [29]. It is believed that there is a relationship between one’s level of knowledge and understanding of mental health disorders and their likelihood to seek help. As such, identifying levels of MHL amongst the general and professional populations has the potential to lead to the implementation of policies and programs aimed at improving MHL. Over the years, MHL has been reconceptualized and now incorporates concepts such as help-seeking attitudes, knowledge of mental health resources, proactive maintenance of good mental health, and awareness of stigmatising attitudes and beliefs [40, 49].

Youth are in a vulnerable period with 75% of all mental health disorders emerging before the age of 24 [45, 50, 91]. Adolescence is a developmental stepping-stone that usually takes place over the ages of 10 and 19, but recent research shows that it may extend to the age of 24 depending on one's puberty [90, 91]. Therefore, we use the term youth, similarly to the United Nations [106], to refer to adolescence and slightly older. Common youth mental health disorders, particularly depression and generalized anxiety, are associated with lasting negative impacts on well-being including the development of adult mental health disorders [45, 46, 64]. Mental health literacy has the potential to act as a buffer against these negative outcomes. Since most youth spend a large portion of their day in classrooms, school-settings are uniquely positioned to facilitate foundational MHL by supporting both staff and students. A variety of strategies can be employed in educational settings. These include creating classroom environments that support open discussion about mental health and combat the stigma surrounding mental disorders [72, 76], fostering healthy relationships through effective communication between school team members and students [25], integrating academic learning with mental and physical well-being [88], implementing adaptations and accommodations for students facing mental health challenges, and promptly guiding students to appropriate professional services, especially during crises [98]. Further defining and cultivating school-based MHL in youth is essential as it establishes a foundational network of engaged educators, peers, and parents, capable of early recognition, ongoing management, and broad prevention of mental health disorders amongst youth. The purpose of this paper is to characterize research efforts on MHL surveys and programs in school-based settings.

## Literature review

### Youth mental health

The incidence of mental health disorders among youth is escalating, with a noticeable increase over the past decade. Roughly 20% of youth will be affected by mental health disorders by 10 years of age [64]. Notably, depression surged by 63% between 2009 and 2017 [105] and anxiety rose by 10% between 2012 and 2018 [77]. This increase in prevalence is magnified by social determinants of health, particularly lower socioeconomic status [113], and sex, as biological females exhibit a significantly higher prevalence of mental health disorders across various studies [94]. More recently the COVID-19 pandemic and resulting public health measures have had a substantial impact on some youth, exacerbating depressive symptoms and deteriorating mental well-being [7, 55, 82, 101]. As restrictions continue to ease and students adjust to a “new normal”, the presence of adult guidance, routines, and support within the classroom environment may play a pivotal role in facilitating a successful transition [83].

### School-based MHL programs

Research indicates that youth may require adult assistance to cultivate MHL and properly identify their mental health challenges [40]. As the adults within school settings, educators and administrators are perceived to be a natural fit for the role of adult mental health guide. Any program that has an objective to improve MHL (whether conceptualized as one component, such as reducing stigma, or multiple, such as reducing stigma and promoting knowledge of resources) can be considered a MHL program. The impact of school-based (and generally educator-led) MHL programs was recently evaluated in a meta-analysis that reported a general increase in MHL levels post-program [3]. More specifically, Perry et al. [80] found that two school-based programs improved student MHL, reducing stigma associated with mental illness. In addition, Ojio et al. [75] found that school-based MHL programs addressed poor MHL (which is the primary barrier to youth help-seeking) resulting in a post-program increase in help-seeking behaviours and overall mental health knowledge. Morgado et al. [69] showed that psychoeducational intervention targeting anxiety literacy helped improve youth’s MHL with a small-to-medium effect size. Counter to the MHL benefits experienced by students, Yamaguchi and colleagues [116] found that educators completing MHL training in preparation for providing school-based programming made broad claims about improvements in their MHL, only to have the improvements found to be of relatively low quality upon further evaluation. This finding is key as MHL programs and resources targeted toward educators and administrators are the foundation upon which school-based MHL programs are built. Gilham et al. [31] found that surveys of teachers’ MHL tend to rely on the definition and measures developed by Jorm et al. [43], though the definition by Kutcher et al. [49, 50] was used in one fourth of the papers reviewed. This finding relevant to educator surveys has not yet been assessed in surveys on students.

### Programs to increase MHL in school settings

Most school-based MHL programs rely on the domino effect initiated by educators completing MHL training—building their own mental health literacy in order to successfully incorporate it into their existing lesson plans. Programs like Headstrong [80], Teen Mental Health First Aid [36, 37], and the Mental Health and High School Curriculum Guide (“The Guide”) [52] all take an educator-led approach to programming. The Guide is a popular Canadian example of educator-led school-based MHL programming that has shown a positive impact on youth MHL. Initially targeting educators, the ultimate goal of The Guide is to increase MHL in both educators and youth students through routine adaptations to the curriculum, which normalizes the inclusion and discussion of mental health concerns. To date, program evaluations have found significant improvement in youth student MHL following engagement with The Guide [70]. The generally positive results of program evaluations for school-based MHL programs have been repeatedly challenged. A previous systematic review found that all evaluations reviewed suffered from at least a moderate risk for bias, with 63% of evaluations at high risk of bias [112]. In addition, two more recent systematic reviews found that the current evidence for the implementation of school-based MHL interventions appears to be methodologically and psychometrically lax [78, 93], demanding increased rigour and regularity of program evaluation for school-based MHL programs—a need that is magnified by the current return to school settings following the recent global COVID-19 pandemic.

### Rationale

As pandemic-related school disruptions recede, youth and educators return to the classroom to re-establish the routines and necessary roles of in-person learning. The integration of MHL into the school environment continues to be an essential part of supporting youth through the trauma of the pandemic, cultivating skills that will carry them through future challenges. Recent scoping reviews have assessed the impact of school-based interventions on youth MHL that is specific to peer-led designs [47], the implementation processes related to school-based mental health services [87], school-based efforts to support youth who specifically cope with a history of trauma [99], and an analysis of the quality of recently-developed school-based MHL interventions [60]. However, to the best of our knowledge, there are no scoping reviews describing the level of documented MHL of school-attending youth and characterizing components of current and previous school-based programs in depth. In order to establish depth of knowledge, we employ a more detailed analysis of school-based MHL interventions by extracting study conceptualization (e.g., MHL definition) in addition to details pertaining to the program and study design. Unique to this scoping review is the description of evidence of effectiveness of the school-based MHL programs, allowing the present review to gather a wide breadth of evidence pertaining to these programs, reporting on the key findings, outcomes, and effectiveness of the programs under investigation.

Our research questions are: (1) What characterizes mental health literacy surveys in school-attending youth? and (2) What are the components of school-based programs to foster MHL in youth?

## Methods

This scoping review was conducted in accordance with the JBI methodology for scoping reviews [81]. Scoping reviews address an exploratory research question by systematically searching, selecting, and synthesizing a wide range of literature to determine the breadth of evidence on a particular topic [81]. They are a type of knowledge synthesis that scopes or maps a body of literature with relevance to time, location, source, method, and origin [54].

### Database search of peer-reviewed literature

**Fig. 1:**
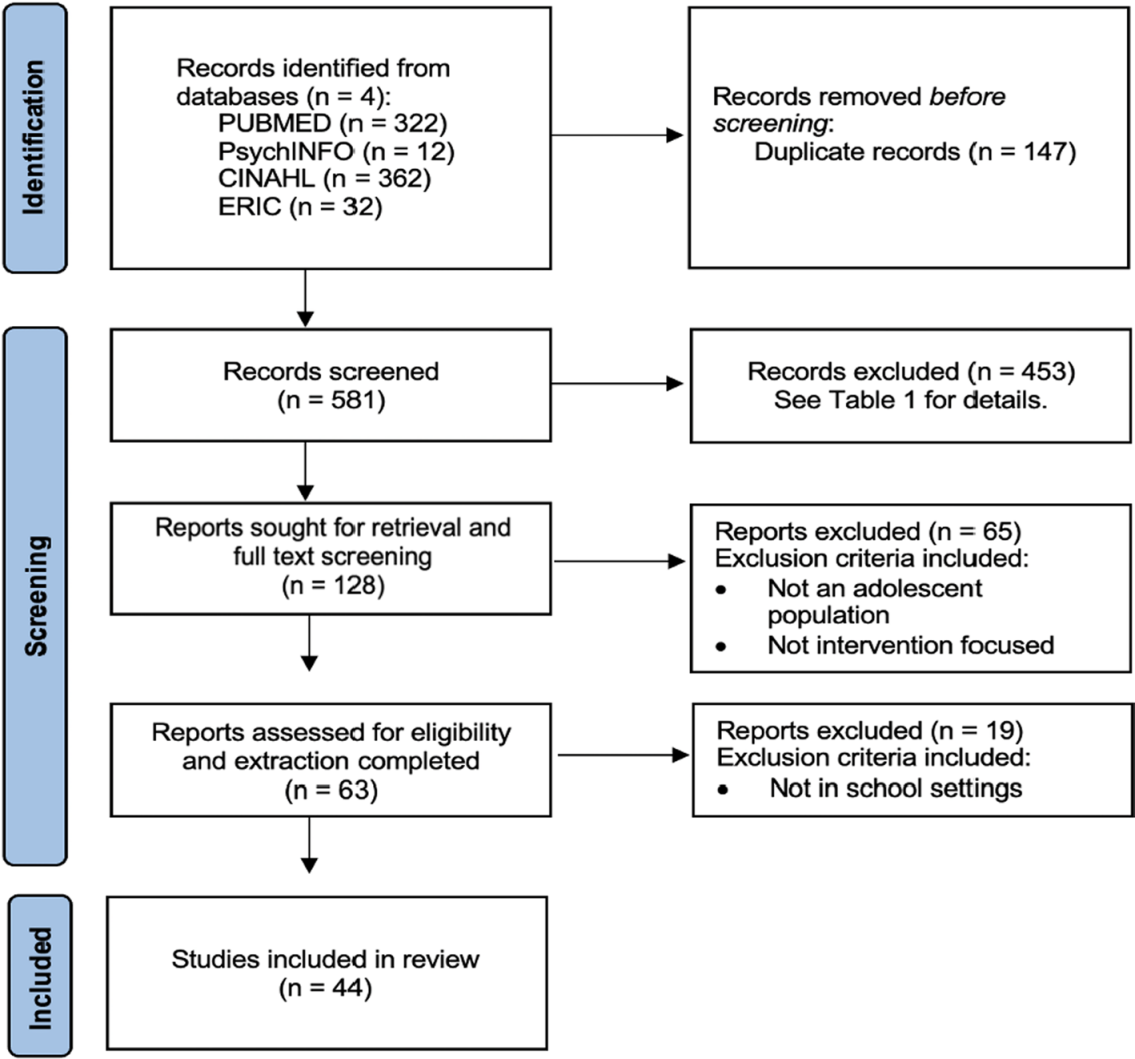
PRISMA flow diagram

A preliminary search of PROSPERO, the Cochrane Database of Systematic Reviews, and JBI Evidence Synthesis was conducted and no current or in-progress scoping reviews on the topic were identified. We then began our database search for articles (see Appendix A for the search strategy). Articles published in English were included. The databases searched include PsychINFO (EBSCO), MEDLINE (PubMed), ERIC, and CINAHL and was conducted on May 4, 2022. The result of the search is reported in a Preferred Reporting Items for Systematic Reviews and Meta-analyses for Scoping Reviews (PRISMA-ScR) flow diagram [67, 103] (see Fig. 1).

The data extracted from relevant published literature is displayed to include details relevant to:

- bibliography (e.g., author and year of publication, country)
- conceptualization (e.g., purpose of study, definition and citation used for defining MHL)
- design (e.g., scale used to measure MHL, study design)
- sample (e.g., size, sex, socioeconomic status, student type, school type), and
- results (e.g., level of MHL in students, key findings of surveys).

Data extracted from included papers is presented in a tabular form, Table 1 reports key findings relevant to the review question. Data was synthesized based on conceptualization and measurement of school-based MHL programs, which were then classified into themes using content analysis. Results were categorized by research questions. A narrative summary accompanies the tabulated data to describe how the results relate to the research question.

**Table 1:** Inclusion and exclusion criteria for review of school-attending youth MHL

Criterion	Inclusion	Exclusion
Time period	2000–2021	Articles outside these dates
Language	English	Non-English articles
Type of article	Open Access, dissertations/theses, peer-reviewed articles including RCTs, quasi-experimental studies, pre-post studies, cluster randomized control trials	Systematic reviews, literature reviews, scoping reviews, meta-analyses, study protocols and grey literature, including government documents
Study focus	School-based MHL programs targeted toward youth including program evaluations reporting outcomes relating to base levels of MHL, stigma, and attitudes towards mental health and help-seeking behaviours	Other specific focus programming, for example substance use prevention, suicide prevention, social-emotional learning interventions, eating disorder prevention, etc
Population/sample	Youth attending secondary school^a^	Children, university students, adults, older adults, individuals with specific disorders
Setting	Secondary schools	Elementary schools, home and community environments^b^

^a^Our search strategy and screening process focused on grade level (secondary school) rather than specific age of the students. Consequently, one study was included which suggests that the end of adolescence is not always defined [90] to capture the full extent of adolescence

^b^One study that measured MHL in youth attending both elementary and secondary school was included to capture the secondary school results

## Results

A database search of the existing literature resulted in the review of 728 studies. After 147 duplicates were removed, we screened 581 articles, deeming 453 irrelevant. This resulted in 128 full-text studies to screen, of which 65 were excluded (see Fig. 1). During extraction, 19 studies were excluded, resulting in 44 included articles. The characteristics of the articles were identified and reported as follows: study continent and year, definition of mental health literacy, baseline level of mental health literacy, types of measures of MHL and measured components, study design, overall program effectiveness, school type, sample size and age range, and differences in MHL by sex. The characteristics pertaining to the baseline MHL, difference in MHL by sex, measurement of MHL, and sample size can be found in Table 2.

**Table 2:** Characteristics of (*N* = 44) school-based mental health literacy surveys of students

Citation	Purpose	Baseline MHL	MHL by sex	Measurement of MHL	Measured component of MHL	Student sample size
Abonassir et al. [1]	Assess MHL among female youth	Moderate		Novel Arabic version of Depression Questionnaire	Recognition	395
Attygalle et al. [6]	Assess MHL amongst youth Sri Lankans	Moderate		Australian National survey on MHL [85]	Recognition & help-seeking	1002
Bjørnsen et al. [8]	Evaluate the efficacy of the MEST program on the positive mental health and mental wellbeing of youth	Moderate	F > M	Mental Health Promoting Knowledge Scale [9]	Program evaluation	357
Bruno et al. [10]	Assess MHL of depression in youth males ages 16 to 18 years of age	High		Friend in Need Questionnaire [11]	Knowledge & attitudes	72
Burns & Rapee [11]	Assess MHL amongst Australian youth	Low	F > M	Friend in Need Questionnaire [11]	Recognition	202
Byrne et al. [12]	Assess MHL of depression and help-giving responses amongst Irish youth by sex	Moderate	F > M	Friend in Need Questionnaire [11]	Knowledge	187
Campos et al. [[14]]	Evaluate the efficacy of a school-based intervention program on youth MHL	Moderate	F = M	MHL Questionnaire [15]	Program evaluation	543
Chisholm et al. [17]	Evaluate the efficacy of a MHL program in combination with a contact intervention on youth MHL	Moderate		Mental Health Knowledge Schedule [26]	Program evaluation	657
Clark et al. [20]	Assess factors impacting help-seeking behaviour for clinical anxiety in Australian youth males	Low		Unspecified vignette	Help-seeking	29
Clark et al. [18]	Assess MHL and help-seeking attitudes related to anxiety amongst Australian youth males	Low		Friend in Need Questionnaire [11]	Help-seeking	1737
Clark et al. [19]	Assess MH stigma related to anxiety amongst Australian youth males	Unspecified		Friend in Need Questionnaire [11]	Stigma	702
Coles et al. [22]	Assess factors impacting MHL of depression and social anxiety amongst US youth	Moderate	F > M	Friend in Need Questionnaire [11] & vignettes [41]	Recognition and help-seeking	1104
Fraser & Pakenham [27]	Evaluate the efficacy of a group psychosocial intervention for youth of a parent with mental illness	Moderate		Unspecified Questionnaire	Program evaluation	44
García-Soriano & Roncero [30]	Assess MHL of obsessive–compulsive disorder amongst Spanish youth	High	F = M	Novel vignettes	Recognition & stigma	102
Haavik et al. [33]	Explore MHL and barriers to help-seeking amongst Norwegian youth by sex	High	F > M	Vignette & questionnaire [41, 42]	Program evaluation	1249
Hart et al. [37]	Evaluate the efficacy of a school-based programme on the MHL of Australian youth	High		Vignette [4, 115] & questionnaires [38, 117]	Program evaluation	1942
Hart et al. [36]	Evaluate the efficacy of a school-based programme on the MHL of Australian youth	Unspecified		Vignette & questionnaire [85]	Program evaluation	475
Katz et al. [44]	Evaluate the efficacy of a school-based intervention combining MHL and Dialectical Behaviour Therapy on the MHL of school-attending Canadian youth	Unspecified	F = M	Resilience Inventory [97], items from Global Portrait of Social and Moral Health for Youth [24], Classroom Supportiveness Subscale from the Child Development Project [16], & Self-Description Questionnaire-General Subscale [61]	Program evaluation	995
Lam [51]	Assess MHL of depression amongst Chinese youth	Low		Depression Literacy Scale [32]	Knowledge	1678
Lanfredi et al. [52]	Evaluate the efficacy of a school-based anti-stigma interventions on MHL amongst Italian youth	Moderate		Knowledge about Mental Illness test (KMI) [111]; Attitudes about Mental Illness questionnaire (AMI) [108]; Mental Illness questionnaire, modified version for schools (WIMI) [111]; revised Attribution Questionnaire (r-AQ-8 version) [111], & California Assessment of Stigma Change (CASC) [23]	Program evaluation	221
Leighton [53]	Assess MHL amongst British youth	Moderate	F > M	Novel vignette-based questionnaire	Recognition	208
Lindow et al. [56]	Evaluate the efficacy of a school-based suicide prevention and MHL promotion amongst US youth	Moderate		Questionnaire (adapted from the General Help Seeking Questionnaire [114] and from a randomized controlled trial of YAM currently being conducted in Stockholm Sweden	Program evaluation	436
Marshall & Dunstan [62]	Assess MHL amongst rural Australian youth	Low	F > M	Friend in Need Questionnaire [11]	Recognition & help-seeking	122
Melas et al. [63]	Assess MHL amongst Swedish youth	Low	F > M	Adapted vignettes [43]	Recognition & help-seeking	426
Milin et al. [65]	Evaluate the efficacy of a school-based MHL intervention amongst youth	Moderate		Questionnaire (developed by authors)	Program evaluation	465
Miller et al. [66]	Evaluate the impact individual educators have on the efficacy of a youth targeted school-based educator-led MHL intervention	High		Adolescent Depression Knowledge Questionnaire [35] & The Reported and Intended Behavior Scale (RIBS) [26]	Program evaluation	6679
Mond et al. [68]	Examine the mental health literacy of adolescent girls concerning bulimia nervosa	Low		Vignette (developed by authors and adapted from mental health literacy protocol) [43]	Help-seeking	522
Morgado et al. [69]	Evaluate the efficacy of a school-based MHL intervention amongst Portuguese youth	Moderate		QuALiSMental (European Portuguese version of the “Survey of Mental Health Literacy in Young People”—Interview Version) [57]	Program evaluation	38
Nguyen et al. [71]	Evaluate the efficacy of a school-based educator-led MHL program amongst Cambodian and Vietnamese youth	Low		Mental Health Knowledge and Attitude Test developed to accompany The Guide [48]	Program evaluation	3275
Ogorchukwu et al. [74]	Assess MHL amongst South Indian youth	Low		Questionnaire (based on the National Survey of MHL and Stigma Youth Boost Survey V5) [86]	Help-seeking, recognition & attitudes	916
Pearson & Hyde [79]	Assess MHL and factors influencing help-seeking behaviours amongst Australian youth	Moderate	F = M	Mental Health Literacy Scale (MHLS) [73]	Knowledge, help-seeking & attitudes	172
Perry et al. [80]	Evaluate the efficacy of a school-based educational MHL intervention amongst Australian youth	Low		Depression Stigma Scale (DSS) [32]; Inventory of Attitudes Towards Seeking Mental Health Services (IASMHS) [59]; Depression Anxiety and Stress Scales, (DASS-21) [58] & Moods and Feelings Questionnaire (MFQ) [5]	Program evaluation	380
Ratnayake & Hyde [84]	Assess MHL, help-seeking, and wellbeing amongst Australian youth	High	F = M	Mental Health Literacy Scale (MHLS) [74]	Help-seeking	32
Riebschleger et al. [89]	Evaluate the efficacy of a school-based educational MHL intervention amongst US youth	Low		Mental Health Literacy Scale (MHLS) [73]; author generated questionnaire	Program evaluation	46
Scott & Chur-Hansen [92]	Assess MHL amongst rural Australian youth	Unspecified		Questionnaire (Mental Health Literacy Questionnaire) & vignette [40]	Recognition	9
Sharma et al. [95]	Assess MHL amongst Indian youth	High	F = M	Novel questionnaire & vignette	Recognition & attitudes	354
Skre et al. [96]	Evaluate the efficacy of a school-based educational MHL intervention amongst Norwegian youth	Low		Unspecified questionnaire	Program evaluation	1070
Thai et al. [100]	Assess MHL amongst Vietnamese youth	Moderate		Mental Health Literacy Scale (MHLS) [73]	Recognition, help-seeking, knowledge & stigma	1075
Tissera & Tairi [102]	Assess MHL for depression, anxiety, and obsessive–compulsive and related disorders amongst New Zealand youth	Moderate		Adapted vignettes [43]	Recognition & help-seeking	114
Trompeter et al. [104]	Assess MHL amongst Salvadorians youth	Low		Vignettes (adapted from the Friend in Need Questionnaire [11] & Mental Health Literacy Questionnaire [43] and created by the authors)	Recognition & attitudes	383
Wang et al. [109]	Assess factors associated with youth help-seeking behaviours related to school-based mental health services	Moderate	F = M	Adolescent Depression Knowledge Questionnaire (ADKQ) [34]	Help-seeking	369
Wang et al. [110]	Explore Asian– and Latinx–American youth perceptions of mental health help-seeking	High		Vignette [43]	Help-seeking	55
Yoshioka et al. [118]	Assess MHL amongst Japanese youth	Low		Vignette [43]	Recognition & help-seeking	311
Zare et al. [119]	To identify the effect of a school-based training intervention on the level of MHL among a sample of female high school students in Iran	Unspecified		Questionnaire & vignette [15]	Program evaluation	220

Amongst the remaining 44 peer-reviewed articles, 27 articles empirically measured MHL not tied to a specific program or intervention, while 17 articles documented program evaluation (15 unique programs).

### Characteristics of mental health literacy surveys in school-attending youth

Interest in youth MHL has grown steadily over the last 20 years, only to surge since 2019 with 45% of the publications reviewed here being published since 2019 (*N* = 20). Globally, Oceanic countries (*N* = 15; 34%) and European countries (*N* = 11; 25%) dominate the literature followed closely by North American (*N* = 9; 20%) and Asian countries (*N* = 8; 18%). In contrast, South American countries entered the dialogue with a single publication in 2021 (*N* = 1; 2%).

### Conceptualization of MHL

Alongside this growth, Jorm and colleagues [43] appear to have established the accepted definition of MHL (”knowledge and beliefs about mental disorders which aid their recognition, management and prevention”) capturing 50% (*N* = 22) of the citations in this review. Jorm and colleagues went on to produce variations of the predominant definition in 2000, 2006, and 2012, which account for 27% (*N* = 12) of the MHL definitions in the present review. Of the remaining MHL definitions, 18% remained unspecified (*N* = 8), while Wei et al. [112] and Kutcher et al. [49] were each cited once (5%).

### Study design and sampling

Study design was largely cross-sectional (*N* = 27; 61%), while the remaining studies included program evaluations (*N* = 13; 30%), longitudinal designs (*N* = 2; 5%), quasi-experimental designs (*N* = 1; 2%), and cluster-randomized trials (N = 1; 2%). More than half of the studies used scale based measures for MHL (*N* = 24; 55%) and the rest of the studies relied on vignettes (*N* = 10; 23%), a combination of scales and vignettes (*N* = 9; 20%), or unspecified tools to measure MHL (*N* = 1; 2%). As this review targets school attending youth MHL, nearly all studies included participants between the ages of 10 and 21-years-old^1^ and 74% of studies were based in secondary/high schools (*N* = 35). The remaining studies varied in participant age, including participants ages 8 to 19 years [44], and school type, including 11% based in middle schools (*N* = 5), 2% in pre-university colleges (N = 1), and 11% did not report a school type (N = 5).^2^ Sample sizes ranged from 9 to 6679 participants.

### Measurement of MHL

Measures for baseline MHL level varied considerably with a total of 28 scales across all articles. The most common measure was the Friends in Need Questionnaire [11] (*N* = 6; 14%) followed by the Mental Health Literacy Scale (MHLS) [73] (*N* = 4; 9%), the Mental Health Literacy Interview [43] (*N* = 5; 11%) as well as three novel author generated measures (7%). The resulting MHL baseline levels were predominantly moderate (*N* = 17, 39%) and low (*N* = 14, 32%), with eight studies finding high MHL baseline levels (18%) and five studies remaining unspecified (11%).

Across the 27 articles 3 that empirically measured youth MHL, it was clearly identified which MHL component was being measured in 16 articles (59%). In the remaining articles, the measured components were identified based on the vocabulary used by the authors to describe their outcome measure (e.g., identify) and how it corresponded to the different MHL component descriptions. The component “recognition” refers to the capacity to identify signs and symptoms of a mental health disorder, “help-seeking” refers to knowing how, when, and where to get help, “knowledge” refers to the understanding of the different mental health disorders, and “stigma/attitudes” refers to the perception held by people with regards to mental health disorders [8, 49]. The most commonly measured component was “recognition” (*N* = 15; 56%), followed by “help-seeking” (N = 13; 48%), “stigma”/”attitudes” (*N* = 8; 30%), and “knowledge” (*N* = 5; 19%). The Friend in Need Questionnaire [11] was the most commonly used (*N* = 5; 19%) questionnaire to measure “recognition” and “help-seeking.”

### Examining gender differences in youth MHL

Amongst studies that assessed differences in MHL by sex (*N* = 15), 53% found that females experienced higher MHL than their male counterparts (*N* = 8) while the remaining 47% found no difference between the sexes (*N* = 7).

### Common components of school-based programs to foster MHL in youth

Across this review, 39% (*N* = 17) of the studies provided an assessment of school-based MHL programs, evaluating the effectiveness of 15 unique MHL interventions—characteristics of which are outlined in Table 3—with more than half being written after 2018 (*N* = 12; 71%). In line with the broader findings of the review, most evaluations were based in Europe (*N* = 6; 35%), closely followed by North America (*N* = 5; 29%), Oceania (*N* = 4; 24%), and Asia (*N* = 2; 12%) and the bulk referenced a version of Jorm et al.’s [43] definition of MHL (can refer to publication years 2006, 2000, and/or 2012; *N* = 10; 59%). Of the remaining studies nearly, a quarter did not specify a definition of MHL (*N* = 5; 29%) and Wei et al. [112] and Kutcher et al. [49] were each cited once (6.25% each). Evaluations overwhelmingly took place in secondary/high school classrooms (*N* = 12; 63%) with the remaining evaluations taking place in an unspecified location (*N* = 3; 16%) or middle schools (N = 3; 16%).

**Table 3:** Characteristics of (*N* = 17) unique school-based mental health literacy programs for students

Article	Program details	Study design	Program effectiveness	Program barriers	Program facilitators
Bjørnsen et al. [8]	Based in positive psychology, *MEST* focuses on an individual’s assets to cultivate good mental health. Delivered by school health services, the program offers voluntary open school seminars, classroom seminars, and smaller group discussions	Analysis of cross sectional cohort data	Minimally effective; significant increase amongst female youth	Flexible nature of program makes evaluation challenging	Adaptable curriculum
Campos et al. [14]	Finding Space for Mental Health: Delivered by trained psychologists, two 90 min sessions delivered at one-week intervals using an interactive methodology (group dynamics, music and videos) to bolster MHL amongst youth	Randomized controlled trial with measures at three time points	Moderately effective; significant increase in MHL in the short-term	Limited training with minimal follow up to solidify learning and knowledge of resources	Not specified
Chisholm et al. [17]	School Space Intervention: A 1-day classroom-based program led by a mental health professional that introduces youth to an individual with mental illness	Pragmatic cluster non-blinded randomized controlled trial with control group and pre- and post-test measures	Moderately effective; intergroup contact proved ineffective and control group significantly more than intervention group	Intergroup contact reduced the impact of the intervention, particularly amongst young students	Not specified
Fraser & Pakenham [27]	Koping Adolescent Group Program (KAP): Three 6-h biweekly peer-led sessions aim to increase MHL and coping skills while fostering peer connectedness amongst adolescents	A treatment and wait list control design with pre- and post-treatment measures, and 8 week follow-up design	Not effective	Not specified	Positive subjective perception of experience, suggesting primed for change
Hart et al. [36, 37]	Teen Mental Health First Aid: Three 75-min classroom-based sessions presented by program trained instructors using a variety of engaging activities to foster MHL and group discussion amongst youth	Cluster-randomized crossover trial with pre- and post-test measures; uncontrolled trial with pre- and post-test measures	Effective overall; effective but less so than in older youth sample	Limited responsivity and student follow up, possibly due to uninteresting content	Sustainable dissemination of model makes increases distribution potential
Katz et al. [44]	Combined MHL and Dialectical Behaviour Therapy (DBT) intervention: A classroom-based, educator-led program targeted toward students in grades 3–12, including nine lessons developing MHL skills and 12 lessons developing DBT skills	Cluster-randomized (by school) design with blind assessors and administration of measures at three time points	Effective overall; significant differences between intervention and control group with large effect sizes	Not specified	Feasible implementation
Lanfredi et al. [52]	Breaking the Silence: Teaching the Next Generation About Mental Illness: Two 2-h active education sessions presented by two clinical psychologists including stories, discussion questions, worksheets, and role playing for help-seeking	Quasi-experimental with partial unmasked randomization to one of the three interventions and measures at two time points	Effective across all interventions; groups led by clinical psychologists had increased MHL	Not specified	Prolonged contact with psychologist reduced mistrust toward specialists
Lindow et al. [56]	Youth Aware of Mental Health (YAM): A school- and curriculum-based mental health promotion and suicide prevention intervention targeted towards youth	Uncontrolled, pre- and post-test design	Effective overall; significant increases in MHL and decreased stigma	Lacks support activities, limiting impact on help seeking with professionals	Not specified
Milin et al. [65]	The Curriculum Guide: A classroom-based, educator-led 6 module (over 6 h) curriculum targeted toward students in grades 11 and 12	Randomized controlled trial with control group and measures administered pre- and post-program implementation	Effective overall	Limited educator training and ongoing support as well as limited resource access	Professionally reviewed curriculum allowing off-the- shelf use; use of collaborative learning techniques
Miller et al. [66]	Adolescent Depression Awareness Program (ADAP): a depression literacy program delivered to high school students by educators	A randomized treatment and wait list control design with pre- and 6 week post-test and 4 month follow up measures	Effective overall	Not specified	Not specified
Morgado et al. [69]	The ProLiSMental psychoeducational intervention: A school-based program run by specialized nurses and including four sessions of 90 min or eight weekly sessions of 45 min that fosters well-being and MHL amongst youth	Pilot study composed of a single-blinded randomization to intervention or wait list control group	Effective overall	Not specified	Self-help approach received positively
Nguyen et al. and Zare et al. [71, 119]	The Mental Health and High School Curriculum Guide (“The Guide”): A classroom-based, educator-led program targeted toward students in grades 8–12 composed of six modules delivered in 60-min sessions	posttest randomized design with control group	Effective overall; small but significant improvements in MHL and stigma with small to medium effect sizes	Variability in quality of lesson plans; limited educator training and ongoing support as well as limited resource access	Web-based materials that support off-the- shelf use; use of collaborative learning techniques
Perry et al. [80]	HeadStrong: a universal curriculum-based program across 5 modules delivered over 5 to 8 weeks or approximately 10 h of class time	Cluster randomized controlled trial with control group and measures administered at three time points	Effective overall; immediately effective (moderate to large effect size) that diminished over time	Lack of supplementary follow up teaching to solidify the base knowledge; difficult research procedures increased attrition	Not specified
Riebschleger et al. [89]	Youth Education and Support (YES) program: A ten-session MHL program led by school social workers for students in grades 5 to 8 with each session lasting 50 min	Pre-post evaluation (no control group)	Effective overall; participants reporting significant improvement in MHL levels	Session times hampered by class days, holidays, etc	Active learning format that is fun and developmentally appropriate
Skre et al. [96]	Mental Health for Everyone program: Based in positive psychology and the theory of salutogenic perspectives, this educator-led school-based, 3-day MHL program targets students ages 13 to 15-years-old	Cluster non-randomized controlled trial with measures administered at two time points	Effective overall	Not specified	Free web-based programming

Study designs included randomized control trial (*N* = 11; 61%), pre-post evaluation (*N* = 5; 28%), quasi-experimental (*N* = 1; 6%), and non-randomized control trial (*N* = 1; 6%). While most evaluations relied on scale-based MHL measures (*N* = 14; 78%), the measures used continued to vary dramatically between studies, with only two studies using the Mental Health Literacy Scale [73] (11%), and two using the Mental Health Literacy Questionnaire [15] (11%). The remaining measures varied by study and three studies did not specify a baseline measure at all (19%).

#### MHL program components

Program implementation across the review was predominantly led by school personnel including educators [44, 65, 66, 71, 80, 96, 119], school social workers [89], and school health services personnel [8, 69]. Mental health personnel including clinical psychologists [14, 17, 52] and program-specific facilitators [36, 37, 56] implemented programs as did peers both in person [27].

Most programs relied on educators and other school health services personnel for program delivery (*N* = 9; 53%). The remaining programs were administered by mental health professionals (*N* = 4; 24%), peers (*N* = 1; 6%), and on a web-based platform (*N* = 1; 6%).

Only 53% of the assessed articles on MHL programs described the format of their programs. Out of those nine articles, it was found that the majority of the programs were classroom-based (*N* = 5), while the remaining programs were either school-based (*N* = 3), or curriculum-based (*N* = 2). One program was delivered in a voluntary fashion.^4^

The delivery of the programs varied across all programs in terms of duration. The training was often offered over the course of days *N* = 1 (1 day); *N* = 1 (3 day) or over multiple shorter sessions (*N* = 10). The number of sessions could vary between two to twenty-one, with the average ranging between three and six sessions. Three programs did not mention the duration of their training.

Two programs, the *Mental Health for Everyone program* and the *MEST*, are based on notions of positive psychology.

#### Barriers and facilitators

Only one program failed to identify program barriers and facilitators. The program barriers mainly fell under the themes of (1) lack of educator training/support, (2) limitation in tools and measures, (3) flexibility of program (e.g., too flexible, difficulty targeting specific topics), and (4) school calendar and class schedule interfered with program delivery. On the other hand, the program facilitators mainly fell into the themes of (1) reviewed materials, making them “off-the-shelf” use, (2) positive feedback and dissemination of knowledge, (3) free web-based programming/materials, and (4) flexibility allowed for adaptability.

### MHL program effectiveness

The reported youth baseline level of MHL varied across the evaluations with most having moderate MHL levels (*N* = 8; 47%), followed by low MHL levels (*N* = 4; 24%), unspecified MHL levels (*N* = 3; 18%), and high baseline MHL levels (*N* = 2; 12%). Most interventions were highly effective (*N* = 11; 65%), with the remaining interventions moderately effective (*N* = 3; 18%), minimally effective (*N* = 2; 12%), and a single ineffective intervention (6%).

## Discussion

The purpose of this review was to both scope the current MHL of school-attending youth and examine the components of current school-based programs targeting this population. We aimed to identify details related to the selected study designs and to the methodological quality of the school-based MHL interventions (i.e., key findings, outcomes, and effectiveness of the programs under investigation).

### Characteristics of mental health literacy surveys in school-attending youth

With the majority of the articles on MHL being published in Oceanic and European countries, it appears as though Westernised countries have been most productive in generating outputs from MHL research on the student population. This is also true for community MHL surveys [39]. On the other hand, developing countries (i.e., African and South American countries) are not as active in the scholarly discourse of student MHL. This contrast points to a lack of diversity in terms of observations and studies. In a previous review on MHL, it was proposed that more developed countries have greater MHL levels than less developed countries [28, 29]. These findings highlight what seems to be an absence of MHL in developing countries, potentially suggesting that the results are subject to mainstream western ideas.

When looking at the widely accepted definition of mental disorders in the Western world, we see a definition reading “a clinically significant behavioural or psychological syndrome or pattern that occurs in an individual and that is associated with present distress, or disability or with significantly increased risk of suffering, death, pain, disability or an important loss of freedom” [4]. Although this definition is used to diagnose mental health disorders in developed countries, it does not include the differing beliefs of various cultures associated with this concept. Indeed, countries such as Nigeria reject the idea that mental suffering is a health disorder [2]. Many African countries include the notion of subjective experiences when explaining “mental disorders” which differs from the objective point of view of westernised countries. Within this argument, we can consider that MHL research may be less prominent in developing countries as their views on mental health differ from the traditional western idea of the concept, potentially making it harder to assess MHL.

#### Definition of MHL

The predominance of Jorm and colleagues [43] definition of MHL within our review presents some limitations to the assessment of MHL in youth. The Canadian Alliance on Mental Illness and Mental Health (2007) report critiqued Jorm’s original MHL conceptualization as omitting facets of MHL knowledge and beliefs that constitute targets for positive mental health promotion in schools. This position resonates, given that none of the surveys reviewed measured all elements of the most comprehensive definition of MHL [40] with a consistent measure. This has also been found in a review of community [39] and educator surveys [31]. This gap in research and practice indicates it may be challenging to measure all elements of MHL in a single study. As aforementioned, the contemporary version of MHL includes components such as help-seeking attitudes, knowledge of mental health resources, proactive maintenance of good mental health, and awareness of stigmatising attitudes and beliefs [40, 49]. Studies that fail to assess these additional elements of MHL, may overlook important barriers to youth help-seeking as well as facilitators for good mental health promotion and prevention for youth populations in education. Our results show that the most frequently evaluated components of MHL in youth are *recognition* and *help-seeking*. Although this is a step in the right direction with regards to having a more complete notion of youth MHL, it would be important to evaluate the remaining components as much to assure effective youth MHL programs.

#### Study design and sampling

The majority of the studies relied on a cross-sectional study design. Considering this type of design is one that allows to collect data at one specific occurrence [13], it has value in measuring baseline MHL. None of the program studies relied on a cross-sectional study, giving certain value to their results as the effects of the program were measured in some form throughout the program and not only at one time point (having baseline data on MHL helps elucidate any program effects).

The age range of the studied population varied between the ages of 8 and 25-year-olds across the included articles. The majority of the studies used a sample of students aged between 10 and 25-years-old. According to Cloutier and team’s paper [21], the studied sample can either be found in the concrete operational stage or the formal operational stage; it is not until the formal operational stage (starting at the age of 12) that an individual is deemed able to think abstractly. As such, students under the age of 12 may have greater difficulty understanding the abstract aspects of mental health disorders and thus render their MHL levels lower. Future studies on the topic should be conducted on a more specific and condensed age group to assure the reliability of the results.

#### Measurement of MHL

Measures for baseline MHL level varied considerably, with the most common measure used being [11] the Friends in Need Questionnaire (2006; *N* = 8; 17%). The Friends in Need Questionnaire employs a vignette method which asks youth respondents to read and comment on what they think is wrong with a fictitious youth. Vignette measures have been commonly used in MHL research as a means of assessing knowledge, beliefs and attitudes of mental health disorders [43]. In the context of youth populations, and particularly for the purpose of assessing “help-seeking efficacy”, which has been cited in contemporary definitions as an important facet of MHL [49], the use of this vignette paradigm presents some limitations. A youth respondent commenting on the pathology presentations depicted through a vignette may be akin to their observations of classmates and the identification of behaviours indicative of emerging mental health disorders in others. However, assessing this observational capacity alone ignores the respondents own insight and their ability to reflect on the state of their own mental health and wellbeing. Studies that seek to accurately assess elements of MHL that might predict help-seeking in youth, or improvements in help-seeking in response to MHL programming, should utilize measures that assess multiple facets of MHL, beyond those captured in the Friends in Need Questionnaire [11].

#### Gender differences and MHL

Across the majority of articles that assessed differences in MHL level in terms of sex, survey results showed that females reported greater levels of MHL than their male counterparts. Research suggests that females experience higher levels of mental health disorders [94], which may indicate that familiarity with mental illness contributes to MHL. Indeed, When analysing the discrepancies between mental health diagnoses across genders, female teenagers tend to experience internalising disorders such as anxiety more often than male teenagers [107]. In turn, in a study on the effects of gender on MHL levels for anxiety disorders amongst teenagers, it was found that females had an overall greater MHL level than their male counterparts [34]. Therefore, females who are exposed to mental illness via peer networks may hold less stigma toward mental illness and therefore have higher MHL. Considering this, future studies could look into the cause of this difference in MHL levels across genders. The findings may align with our hypothesis and as such, MHL programs may need to be formatted in a way that targets teenage males and attends to their specific lower MHL levels.

### Components of school-based programs to foster MHL in youth

Overall, the majority of the school-based programs aimed to improve teenage students’ knowledge on mental health disorders and bolster their notion of interventions and available support. The program delivery varied across the programs, but they were mostly educator-led, classroom-based, and delivered over multiple sessions.

Amongst the reviewed MHL programs, all but one explicitly reported effectiveness in MHL outcomes. However, none of the programs relied on the same design or delivery method. Of course, the majority were primarily led by educators, class-based, and training was given over the course of several sessions; however, none appeared to use the same numbers of sessions or methods. In addition to the differing types of programs, it was found that none of the studies used the same evaluation methods to investigate the strength of their program or relied on the same sample size. Having inconsistent protocol evaluations and sample sizes brings to question whether or not the same results would be obtained if the same methods would be used.

As one program relied on the use of mental health experts to inform the program, it seems contradictory that one of the main identified program barriers was a lack of educator training or support and that one of the main identified program facilitators was accessible materials. Since MHL is a topic associated with mental health, one would assume that any efforts made to improve it would be based on knowledge translated by experts in the field. However, it appears that only one program in this review relied on said experts. This finding points to the importance of questioning the validity of the material used in the reviewed programs and further encourages future researchers to look into the efficacy of programs that rely or don’t rely on material provided by experts in the field of mental health. Furthermore, it is intriguing how educators who were actively involved in program design, felt a lack of support, but also appeared to be satisfied with the facility they experienced to find material. Considering the contrasting perspectives of level of support, it may be important to pay attention to the effectiveness of the results and further investigate whether better mental health expert support would yield higher levels of MHL among students. Interestingly, a previous review on educator MHL by Gilham et al. [31] found that educators generally had low levels of MHL themselves. This further suggests that teachers may actually be ill-equipped to educate in-school youth on MHL and that the use of a valid way of measuring levels of MHL is necessary in order to truly identify levels of MHL. In line with these contradictory claims, the remainder of the identified barriers and facilitators are opposites (i.e., (1) flexibility of programs: either it makes it hard to target specific programs or it allows for adaptability; (2) materials: either hard to find or free online material).

When reviewing these results, all programs report some positive outcomes. To continue to identify ‘what works’ for school-based MHL programming, funding for research on MHL programs for youth should be increased so that long-term evaluation of mental health programs for youth can be performed. The long-term evaluations will enable assessment of the sustainability of these reported positive outcomes.

## Limitations and future directions

This review was limited by the search strategy. Our search strategy was developed to identify all peer-reviewed literature that empirically measured MHL in youth students via surveys and any other methods of assessing MHL (e.g., teacher informant report) were not included. Thus, our findings are based on evidence garnered from school-based surveys of MHL and may not be applicable to the general population.

None of the articles reviewed measured all elements of the most comprehensive definition of MHL [40] with a consistent measure, suggesting that it may be challenging to measure all elements of MHL in a single study. Thus, while we are unable to identify levels of MHL in youth, we are able to highlight this gap in research and practice.

Future research on school-based MHL surveys should assess and report factors that influence individual’s ability to become mental health literate that can help enable appropriate design of surveys (i.e., measuring determinants of, as well as level of, MHL) which can in turn, inform future implementation of MHL interventions and programs in school settings. For example, international students from newcomer families may conceptualize MHL in a linguistically and/or culturally specific way. Finally, a synthesis of MHL levels across different student characteristics (e.g., grade level, school type, experience with health curriculum that could influence literacy) would improve generalizability and add breadth to the current evidence base.

## Conclusion

In the present study, we aimed to identify what characterizes mental health literacy surveys in school-attending youth and to uncover the components of school-based programs that foster MHL. Our review showed that most school-attending youth have moderate baseline levels of MHL, with females experiencing higher levels than their male counterparts. School-based MHL programs are relatively unified in their definition and measures of MHL, using closed-ended scales, vignettes, or a combination of the two to measure youth MHL. This research should in turn generate information that can be used to create policies in the educational system to implement MHL education in the curriculum. As seen in this scoping review, the majority of the programs in place have yielded improved MHL levels amongst their participants. Providing access to MHL education to the youth through school curriculums would provide them with improved knowledge and recognition for mental health disorders and hopefully reduce potential stigma and thus encourage help-seeking. Accordingly, this could reduce the current rates of mental health disorders amongst our youth and in turn improve their quality of life and reduce the demand on the health and psychological services that struggle to offer services.

To address limitations of the field, a key task will be to develop a standardized tool for assessing MHL levels for youth. Identifying the base levels of MHL amongst school-attending youth promotes the development of targeted programs and reviewing the alignment with program components would allow researchers to build on what works, alter what does not, and come away with new ways to approach these complex challenges, ultimately advancing knowledge of MHL and improving levels of MHL.

## Data Availability

No datasets were generated or analysed during the current study.

## Appendices

### Appendix A

**Table:**

#	Query	Search Details	PUBMED	PsychINFO	CINAHL	ERIC
5	#1 AND #2 AND #3 AND #4 AND #5	(“mental health” [Title/Abstract] OR “mental hygiene” [Title/Abstract] OR (“mental health” [Subject] OR “mental hygiene”[Subject])) AND (“literacy” [Title/Abstract] OR “illiteracy” [Title/Abstract] OR “illiterate” [Title/Abstract] OR “literate” [Title/Abstract]) AND (educat* OR teach* [Title/Abstract] OR educat* OR teach* [Subject]) AND (intervention OR program OR train* [Title/Abstract] OR intervention OR program OR train* [Subject])	322	12	362	32
4	(literacy[Title/Abstract] OR illiteracy[Title/Abstract] OR illiterate[Title/Abstract] OR literate[Title/Abstract]) OR (literacy[Subject] OR illiteracy[Subject] OR illiterate[Subject] OR literate[Subject)	“literacy”[Title/Abstract] OR “illiteracy”[Title/Abstract] OR “illiterate” [Title/Abstract] OR “literate”[Title/Abstract]	29,462	9.557	17,312	13,737
3	(“mental health”[Title/Abstract] OR “mental hygiene”[Title/Abstract]) OR (“mental health”[Subject] OR “mental hygiene”[Subject])	“mental health”[Title/Abstract] OR “mental hygiene”[Title/Abstract] OR “mental health”[Subject] OR “mental hygiene”[Subject]	187,957	47,632	104,848	9958
2	(educat* OR teach* [Title/Abstract] OR educat* OR teach* [Subject])	(educat* OR teach* [Title/Abstract] OR educat* OR teach* [Subject])	785,714	122,658	398,450	496,139
1	(intervention OR program OR train* [Title/Abstract] OR intervention OR program OR train* [Subject])	(intervention OR program OR train* [Title/Abstract] OR intervention OR program OR train* [Subject])	1,570,104	153,807	836,907	236,345

Limited to French and English, no time limit imposed. Date of search: May 4, 2022

## Footnotes

1 The inclusion criteria for papers were based on school level, not on student age. One study [1] is based in Saudi Arabia, and reported results from a ‘governmental secondary school’ with student age ranging from 15 to 20 years old. Thus, our report of student age in this paper may seem higher than is typically expected.

2 The total school type exceeds the number of articles (N = 44) because some studies took place in more than one school type.

3 The articles focused on program evaluation weren’t considered here as their analysis mostly focused on program effectiveness.

4 There were nine programs captured; *N* exceeds nine because one program was both school- and classroom-based.
